# Altered peripheral *CRY1* gene expression may contribute to both organic and functional gastrointestinal disease

**DOI:** 10.1113/EP092725

**Published:** 2025-08-06

**Authors:** Sophie Fowler, Gemma M. Paech, Emily C. Hoedt, Jennifer C. Pryor, Cheenie Nieva, Jessica K. Bruce, Prema M. Nair, Guy D. Eslick, Simonne Sherwin, Peter Pockney, Nicholas J. Talley, Grace L. Burns, Simon Keely

**Affiliations:** ^1^ School of Biomedical Sciences & Pharmacy, College of Health, Medicine and Wellbeing University of Newcastle Newcastle New South Wales Australia; ^2^ NHMRC Centre of Research Excellence in Digestive Health University of Newcastle Newcastle New South Wales Australia; ^3^ Immune Health Research Program Hunter Medical Research Institute New Lambton Heights New South Wales Australia; ^4^ School of Medicine & Public Health, College of Health, Medicine and Wellbeing University of Newcastle Newcastle New South Wales Australia; ^5^ Department of Respiratory and Sleep Medicine John Hunter Hospital Newcastle New South Wales Australia; ^6^ Medical School University of Western Australia Crawley Western Australia Australia

**Keywords:** circadian rhythms, fatigue, inflammatory bowel disease, irritable bowel syndrome, sleep

## Abstract

Gastrointestinal conditions such as irritable bowel syndrome (IBS) and inflammatory bowel diseases (IBD) are characterized by alterations in physiological and immune functions. Given the circadian clock influences gastrointestinal physiology and immunity, we hypothesized that the peripheral circadian clock is altered in these patients and might contribute to immune activation associated with IBD and IBS. To investigate this, RNA was extracted from whole blood obtained from control subjects (*n* = 29), IBD (*n* = 40 ulcerative colitis, *n* = 38 Crohn's disease) and IBS (*n* = 38) participants to investigate peripheral clock gene expression via quantitative PCR. A linear regression model was used to assess the impact of the time of blood collection on clock gene expression. Self‐reported data regarding fatigue and sleep indices were compared between patients and control subjects. Gene expression analysis revealed variations in the peripheral circadian system between IBD, IBS and control subjects. The core clock gene *CRY1* had higher relative expression in IBS (*p* = 0.031) and ulcerative colitis patients (*p* = 0.042) compared with control subjects. Patients with gastrointestinal disease demonstrated poorer quality sleep (IBS *p *< 0.001, UC *p = *0.025 and CD *p = *0.007) and more troublesome sleep (IBS *p *< 0.001, UC *p = *0.002 and CD *p = *0.009) compared with control subjects. These data suggest a role for *CRY1* gene expression in patients experiencing fatigue and highlight a link between circadian dysregulation and the pathophysiology of intestinal disease.

## Abstract (highlights)

- **What is the central question of this study?**
  Is peripheral circadian clock gene expression altered in patients with inflammatory bowel diseases and irritable bowel syndrome?
- **What is the main finding and its importance?**
  We found altered *CRY1* gene expression in patients with ulcerative colitis and irritable bowel syndrome, which was linked to increased fatigue. This indicates that circadian gene expression might be altered in patients with gastrointestinal disease and could be involved in the increased fatigue experienced by these patients.

## 1 INTRODUCTION

Fatigue and sleep disruption are increasingly reported as bothersome extra‐intestinal manifestations of gastrointestinal (GI) conditions, including the irritable bowel syndrome (IBS) and inflammatory bowel diseases (IBD) (Burns et al., 2021). IBS is a disorder of gut–brain interaction (DGBI) characterized by chronic abdominal pain, associated with changes in stool consistency and a lack of evidence of structural disease (Drossman et al., 2006). The aetiology of this disease is poorly understood; however, generally, IBS is characterized by visceral hypersensitivity and intestinal motor abnormalities. In a subset of IBS patients, there is evidence of low‐grade intestinal microinflammation, including increased activated mast cells (Walker et al., 2009) and increased circulating gut‐homing T cells (Burns et al., 2019). In contrast, IBD are well‐established chronic inflammatory diseases, with the two major subtypes of Crohn's disease (CD) and ulcerative colitis (UC). CD is characterized by inflammation anywhere along the GI tract and full‐thickness inflammation of the affected segments of the bowel wall, whereas UC involves inflammation in the mucosa and submucosa of the colon and rectum (Ha & Khalil, 2015). The exact pathophysiology of IBD remains unclear, but lifestyle factors (Sonnenberg, 1990), genetics and other environmental influences are thought to play a role (Jarmakiewicz‐Czaja et al., 2022).

There is evidence of a bidirectional relationship between the gut and the circadian system, with both acute and chronic GI symptoms being associated with sleep disturbances (Kim et al., 2013; Nojkov et al., 2010; Saeed & Galal, 2017). For instance, although distinct conditions, patients with both IBS and IBD commonly report sleep disturbances (Keefer et al., 2006), and increased severity of disease is associated with reduced sleep quality (Tu et al., 2017). Circadian rhythms are cyclic patterns of physiological, behavioural and molecular events that occur over a ∼24 h period. These rhythms are controlled by the suprachiasmatic nucleus in the hypothalamus, the master pacemaker that governs peripheral clocks (Plautz et al., 1997). The sleep–wake cycle, digestion and absorption of nutrients (Segers & Depoortere, 2021; Taleb & Karpowicz, 2022), epithelial barrier function, motility and microbial activity (Rosselot et al., 2016) are influenced by the circadian system, and circadian rhythms play a crucial role in regulating immune function (Dimitrov et al., 2009; Litvinenko et al., 2005; Scheiermann et al., 2012). Furthermore, there is a strong correlation between diagnosis with a GI disease and self‐reported poor sleep quality (Koloski et al., 2021; Withrow et al., 2021; Yamawaki et al., 2014; Yang et al., 2020). Individuals performing rotating shift work (Nojkov et al., 2010) or recovering from jet lag report increased incidences of GI symptoms, including abdominal pain, constipation and diarrhoea (Caruso et al., 2004; Knutsson & Bøggild, 2010), linking chronodisruption to altered GI function. Studies have also reported a higher prevalence of GI problems, including abdominal pain and distension, in patients with chronic insomnia (Bhaskar et al., 2016; Hyun et al., 2019; Taylor et al., 2007). This suggests that altered sleep patterns can aggravate GI symptoms, and subsequently, improved sleep quality might help with symptom management; however, this has been underexplored to date.

The circadian system consists of transcription–translational feedback loops of genes including brain and muscle arnt‐like protein 1 (*BMAL1*), circadian locomotor output cycles kaput (*CLOCK*), period (PER) 1–3, cryptochrome (*CRY*) 1–2 and nuclear receptor subfamily 1 group D member 1 (*NR1D1*) and 2 (*NR1D2*), collectively known as clock genes. This system involves a negative core feedback loop, which is the main driver of circadian rhythms (Mrosovsky et al., 2001; Shearman, 2000), and an auxiliary feedback loop, which helps to maintain the robustness of the circadian system (Bae et al., 2000, 1998; Preitner et al., 2002). These rhythms also provide internal temporal organization to ensure central coordination of internal changes. Clock gene expression has also been correlated with sleep quality (Archer et al., 2008; Arnardottir et al., 2014; Lech et al., 2016; Reszka et al., 2013; Takimoto et al., 2005), and individuals who are resistant to sleep loss exhibit reduced amplitude of clock gene expression (Arnardottir et al., 2014). Importantly, peripheral clock gene transcript expression was shown to display a circadian rhythm, which was correlated with plasma melatonin secretion (an indicator of circadian phase; Lewy et al., 1999). Therefore, assessment of peripheral indicators of circadian disruption might be an effective and minimally invasive method for studying the impact of circadian systems on GI disease, circumventing ethical and clinical considerations in obtaining GI biopsies for such studies.

Given the evidence that central circadian disruption has a peripheral signature and the fact that many functions within the GI tract are controlled by the circadian system, disruptions to this system are likely to be involved in disease and loss of homeostasis. We hypothesized that the peripheral circadian clock is altered in patients with chronic GI conditions, and this might contribute to symptom burden and disease course in these patients. As such, the aim of this study was to determine whether there were alterations in peripheral clock gene expression in patients with both functional and organic chronic GI conditions and whether this was linked to the increased fatigue and sleep disturbances reported by these patients.

## 2 MATERIALS AND METHODS

### 2.1 Participant cohort and questionnaires

This was a retrospective feasibility study, with consenting community participants and outpatients initially recruited from the John Hunter Hospital Gastroenterology Outpatient Clinic (Newcastle, NSW, Australia) and selected from the Digestive Health Biobank (www.digestivehealth.org.au) to participate in the study. This study was undertaken with approval from the Hunter New England Human Research Ethics Committee (reference 2020/ETH01635 and 2020/ETH03303). The study was conducted according to the Declaration of Helsinki (2024), with the exception of clause 35. Participants were required to be aged between 18 and 83 years. Community participants were selected without GI disease or symptoms as a control population, and IBS participants met the ROME IV diagnostic criteria (Lacy & Patel, 2017).

Medical history, medication records and demographic data were collected with a medical interview for outpatients, and all participants answered the validated ‘Digestive Health and Wellbeing’ survey (Potter et al., 2018) to provide information on their symptom profiles, sleep habits and fatigue levels. Fatigue was defined as reporting fatigue several times a week or daily over the past year, and ‘bothersome fatigue’ was defined by answers of ‘extremely’ and ‘severely’ when asked, ‘How bothersome was this in the past year?’. These qualitative responses were then used to assess gene expression in those experiencing bothersome fatigue in comparison to those without. To assess the prevalence of sleep problems within our cohort, participants were screened with the validated Shortened Sleep Condition Indicator Questionnaire (Espie et al., 2014). Answers were converted into scores from zero to four, with a lower score (in the range zero to two) indicating poorer sleep quality, according to Luik et al. (2019). Where available and/or required, patient data were reviewed by a gastroenterologist to confirm self‐reported diagnoses.

### 2.2 Blood sampling

Whole blood samples (6 mL) were collected in EDTA blood collection tubes (BD Biosciences, NJ, USA), the time of collection was recorded, and blood was stored at −80°C prior to RNA isolation. Samples were collected and stored from 2013 to 2022.

### 2.3 RNA isolation

RNA was extracted from human whole blood samples using the simplyRNA blood kit and automated Maxwell RSC 48 system (Promega Corporation, Madison, WI, USA) following the manufacturer's instructions using a total volume of 5 mL of whole blood. All samples were quantified using the Nanodrop 1000 spectrophotometer (ThermoFisher, MA, USA) to determine the integrity and concentration of the extracted RNA. To confirm the integrity of RNA, gel electrophoresis was performed to visualize RNA integrity on a 1% agarose gel. The gel was visualized using an ultraviolet transilluminator chamber, and the RNA was considered intact if bands for 18S and 28S ribosomal RNA were present.

### 2.4 Quantitative PCR

Complementary DNA was constructed using iScript cDNA Synthesis Kit (Bio‐Rad) according to the manufacturer's protocol. Quantitative PCRs were performed using the iTaq Universal SYBR green supermix (Bio‐Rad) on a QuantStudio 6 Flex System PCR machine (BioRad). Relative expression values were determined using the comparative *Ct* (ΔΔ*Ct*) method, with *ACTB* (β‐actin) as the housekeeping gene. Primer sequences (Integrated DNA technologies, Coralville, IA, USA) are described in Table 1.

**TABLE 1:** Primers for quantitative PCR.

Primer	Primer sequence	Annealing temperature (°C)
*ACTB*	Forward: 5′‐GGAGAAAATCTGGCACCACA‐3′ Reverse: 5′‐AGAGGCGTACAGGGATAGCA‐3′	All temperatures
*BMAL1*	Forward: 5′‐GGAAAAATAGGCCGAATGAT‐3′ Reverse: 5′‐TGAGCCTGGCCTGATAGTAG‐3′	60
*CLOCK*	Forward: 5′‐TGCGAGGAACAATAGACCCAA‐3′ Reverse: 5′‐ATGGCCTATGTGTGCGTTGTA‐3′	60
*CRY1*	Forward: 5′‐TTGGAAAGGAACGAGACGCAG‐3′ Reverse: 5′‐CGGTTGTCCACCATTGAGTT‐3′	60
*CRY2*	Forward: 5′‐GGTGTGGAAGTAGTGACGGAG‐3′ Reverse: 5′‐GTAGGTCTCGTCGTGGTTCTC‐3′	63.5
*PER1*	Forward: 5′‐AGTCCGTCTTCTGCCGTATCA‐3′ Reverse: 5′‐AGCTTCGTAACCCGAATGGAT‐3′	60
*PER2*	Forward: 5′‐CTTCAGCGATGCCAAGTTTGT‐3′ Reverse: 5′‐CGGATTTCATTCTCGTGGCTTT‐3′	63.5
*PER3*	Forward: 5′‐TCGTTCTCTGATGGTTGCCA‐3′ Reverse: 5′‐GAAATCTTCCGGCTCCAGGG‐3′	65
*NR1D1*	Forward: 5′‐GCAAGGGCTTTTTCCGTCG‐3′ Reverse: 5′‐CATCCGCTGCTTCTCTCGTT‐3′	60
*NR1D2*	Forward: 5′‐CGTTGACTGCATCTCCCTGA‐3′ Reverse: 5′‐ACAGAACTCCTACCACAAAGGA‐3′	60

### 2.5 Statistical analysis

Statistical analyses and graphs were produced using GraphPad Prism 9 (Graphpad Software Inc., La Jolla, CA, USA) and RStudio (Posit PBC, Boston, MA, USA). Significant outliers were assessed using Grubb's test, and normality of distribution was assessed using the Shapiro–Wilk test. Fisher's exact test was used to determine associations between two variables. The variance of the means was tested with a parametric or non‐parametric ANOVA based on normality assessment. A linear regression model was used to assess the effects of time of blood collection, sex, age and body mass index (BMI) on clock gene expression. A dissimilarity matrix was computed from the gene expression data using Euclidean distance, which then underwent principal components analysis. Gene expression data were Euclidean transformed (AmpVis2) before applying principal coordinates analysis (PCoA). All figures and statistical analyses are presented as the median (interquartile range), and *p *< 0.05 was considered statistically significant.

## 3 RESULTS

### 3.1 Demographic details of study cohort

Twenty‐three consenting community participants and 122 outpatients were included in this study, consisting of 29 control subjects, 40 participants with UC, 38 with CD and 38 with IBS. Blood samples were collected from 07:50 to 16:20 h throughout the day. The general characteristics of the participants investigated showed no significant differences in BMI, alcohol use, proton pump inhibitor use and sex between the control or GI disease groups. The IBS patients were significantly older than CD patients (*p = *0.049), and patients were more likely to have reported current or previous smoking history compared with control subjects, with the prevalence being significantly different between UC (*p *< 0.001), CD (*p <* 0.001) and the IBS cohort (*p = *0.002) (Table 2).

**TABLE 2:** Summary of baseline characteristics of cohort.

Characteristics	Control	UC	CD	IBS
Number of participants	29	40	38	38
IBS subtype: IBS‐D/IBS‐C/IBS‐M/IBS‐U, *n*				10/4/13/8
Age at baseline, median (IQR)	48 (65.0–27.0)	48 (63.5–29.0)	37 (53.0–27.0)	48 (64.5–35.0)
Female, *n* (%)	19 (65.5)	19 (47.5)	21 (55.3)	19 (50.0)
BMI, median (IQR)	23.96 (29.12–22.43)	25.87 (30.69–23.38)	27.78 (32.13–24.16)	27.41 (31.18–24.16)
Current PPI use, *n* (%)	1 (3.4)	4 (10.0)	3 (7.9)	6 (15.8)
Ever smoked, *n* (%)	2 (6.9)	22 (55.0)	18 (47.4)	16 (42.1)
Currently drink alcohol, *n* (%)	16 (55.2)	22 (55.0)	21 (55.3)	16 (42.1)

*Note*: Data were missing from all categories.

Abbreviations: BMI, body mass index; CD, Crohn's disease; IBS, irritable bowel syndrome; IBS‐C, IBS constipation subtype; IBS‐D, IBS diarrhoea subtype; IBS‐M, IBS mixed subtype; IBS‐U, IBS unknown subtype; PPI, proton pump inhibitor; UC, ulcerative colitis.

### 3.2 Participants with GI disease report poorer sleep quality

**FIGURE 1:**
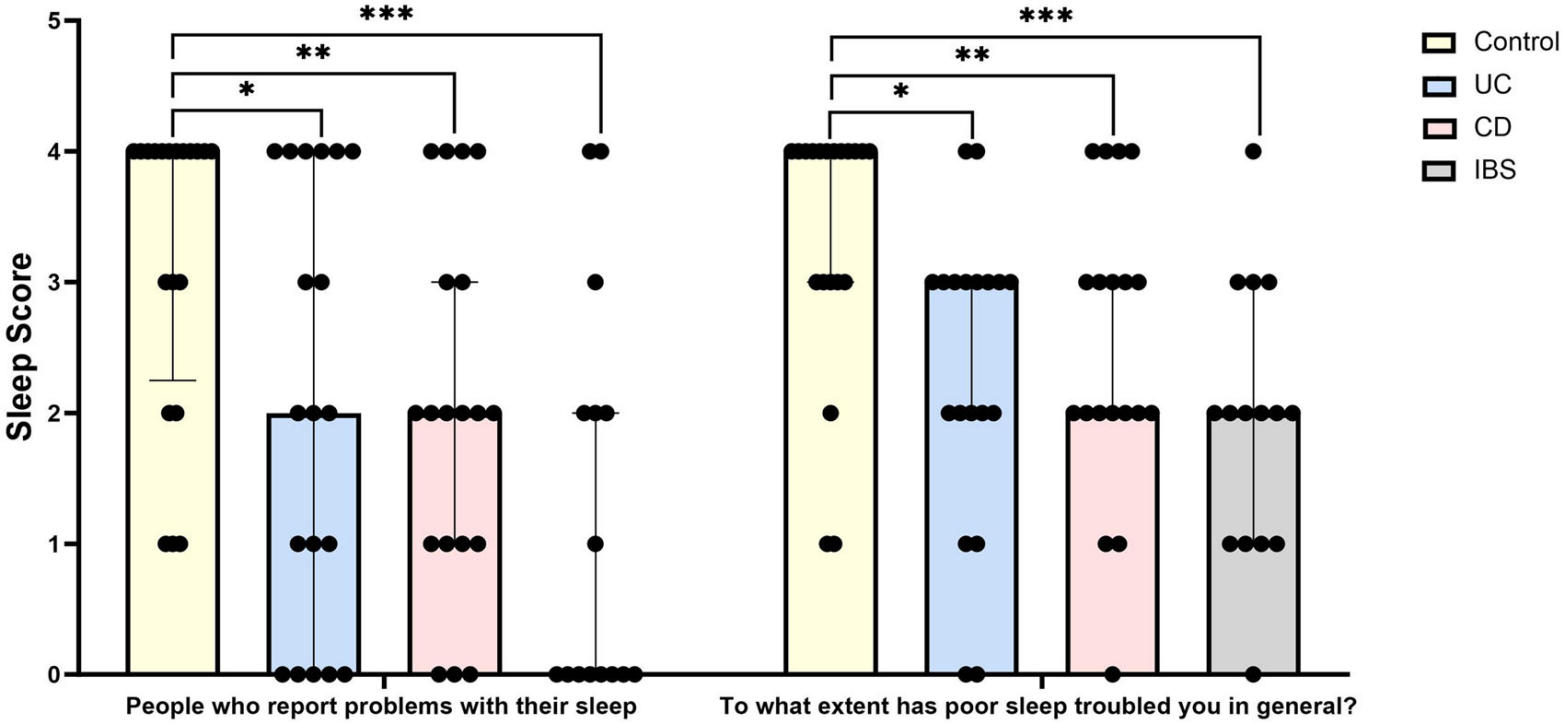
Sleep score from the sleep condition indicator questionnaire. The shortened sleep condition indicator was used to screen for insomnia in control subjects, UC, CD and IBS participants. Participants answered, ‘How many nights a week do you have a problem with your sleep?’ and ‘To what extent has poor sleep troubled you in general?’, and each answer was assigned a score from zero to four. A higher sleep score corresponds to better sleep. *n* = 20 control subjects, *n* = 19 UC, *n* = 19 CD and *n* = 15 IBS. Data are presented as the median (interquartile range) and analysed by multiple Mann–Whitney *U*‐tests with multiple comparisons. *p < 0.05, **p < 0.01 and ***p < 0.001. Abbreviations: CD, Crohn's disease; IBS, irritable bowel syndrome; UC, ulcerative colitis.

Participants with IBS, UC and CD demonstrated significantly lower sleep scores when asked, ‘How many nights a week do you have a problem with your sleep?’, compared with control subjects (IBS *p *< 0.001, UC *p = *0.025 and CD *p = *0.008). When asked, ‘To what extent has poor sleep troubled you in general?’, IBS, UC and CD patients also demonstrated significantly lower sleep scores than control subjects (IBS *p *< 0.001, UC *p <* 0.001 and CD *p = *0.002; Figure 1), demonstrating that patients with GI disease report poorer sleep than control subjects. There was no difference in the sleep score of patients with IBD compared with IBS.

### 3.3 CD participants report increased bothersome fatigue

**FIGURE 2:**
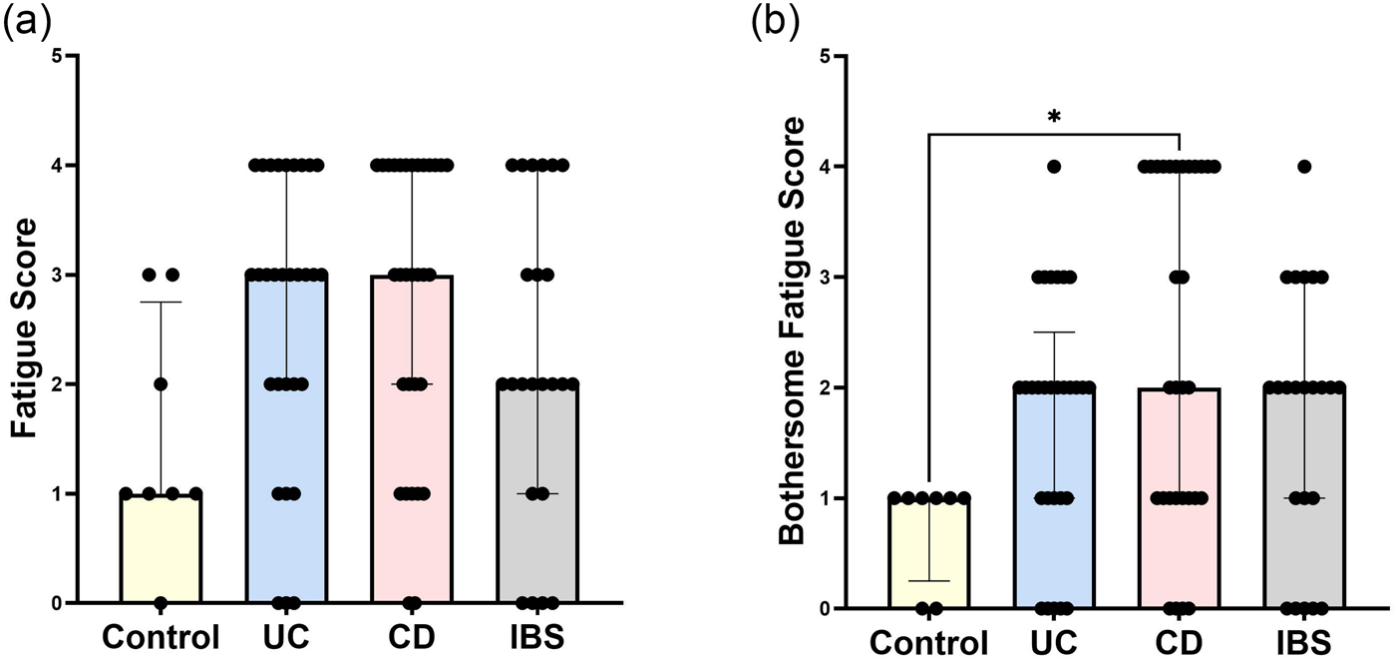
Self‐reported fatigue in control subjects, UC, CD and IBS patients. Participants answered questions relating to fatigue and how it affects them. Participants reported how often they experience fatigue (a) and how often they experience bothersome fatigue (b), and each answer was assigned a score from zero to four. *n* = 8 control subjects, *n* = 29 UC, *n* = 30 CD and *n* = 23 IBS. Data were analysed by the Kruskal–Wallis test. *p < 0.05. Abbreviations: CD, Crohn's disease; IBS, irritable bowel syndrome; UC, ulcerative colitis.

Given that fatigue is frequently reported as a bothersome extra‐intestinal symptom in GI disease (Burns et al., 2021), participants answered: ‘How often has fatigue occurred in the last year?’ (measuring frequency) and ‘How bothersome was this in the past year?’ (assessing impact on quality of life). There was no significant difference between groups for those who reported experiencing fatigue (Figure 2a; Table A1). However, CD (but not UC or IBS) patients reported more bothersome fatigue compared with control subjects (*p = *0.015; Figure 2b). This suggests that there is no difference in the frequency of experiencing fatigue, but the perception and impact of fatigue are more pronounced in CD patients.

### 3.4 *CRY1* gene expression is increased in IBS and UC patients compared with control subjects

**FIGURE 3:**
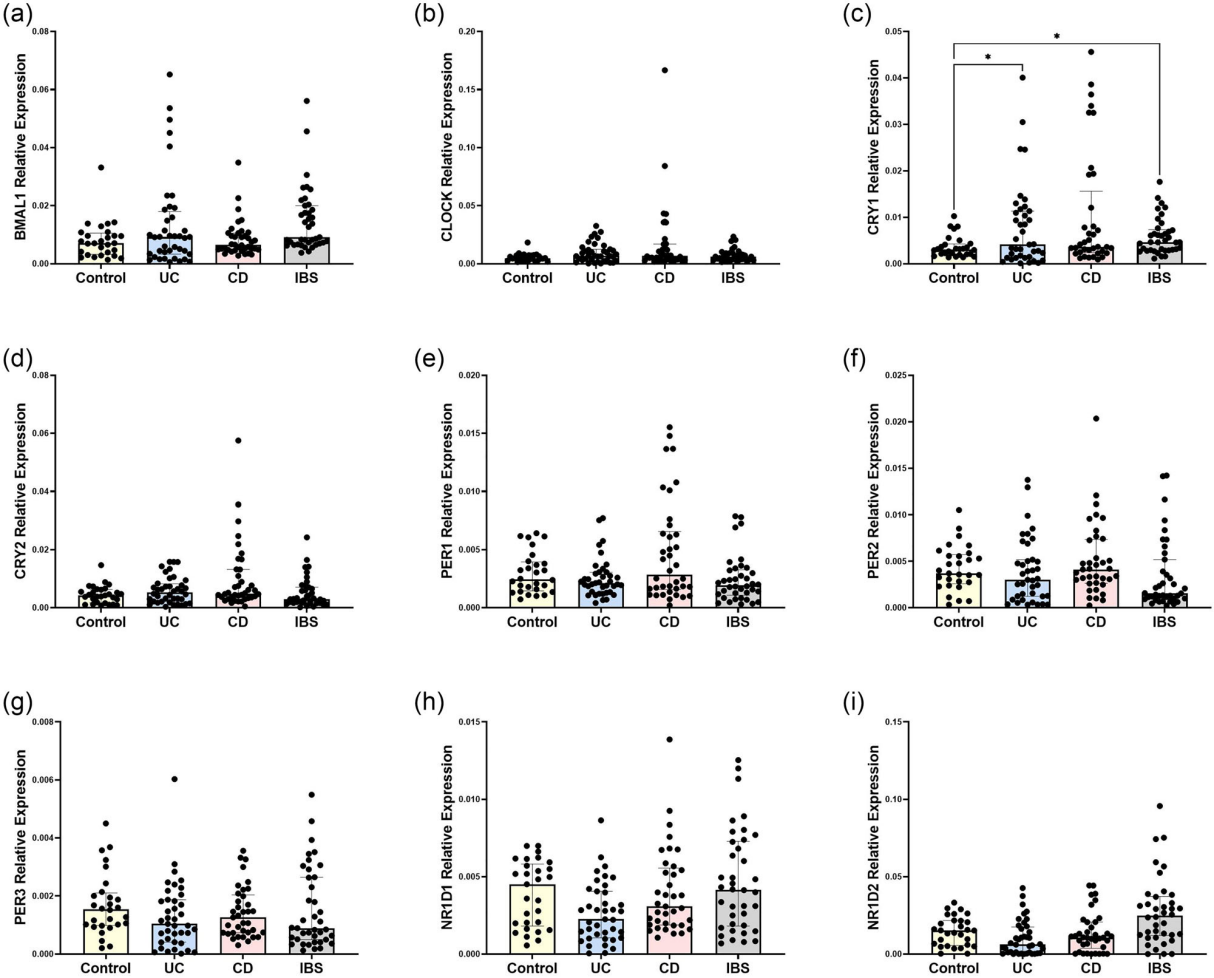
Analysis of circadian clock gene expression profiles in control subjects and gastrointestinal disease patients. Circadian rhythmicity was determined from a cohort of control subjects, UC, CD and IBS patients. Quantitative PCR was used to analyse relative expression of *BMAL1* (a), *CLOCK* (b), *CRY1* (c), *CRY2* (d), *PER1* (e), *PER2* (f), *PER3* (g), *NR1D1* (h) and *NR1D2* (i) relative to the β‐actin housekeeping gene. *n* = 29 control subjects, *n* = 40 UC, *n* = 38 CD and *n* = 39 IBS. Data are presented as the median (interquartile range) and analysed by a linear regression model to account for the effects of time of blood collection, sex, age and body mass index. *p < 0.05. Abbreviations: CD, Crohn's disease; IBS, irritable bowel syndrome; UC, ulcerative colitis.

**FIGURE 4:**
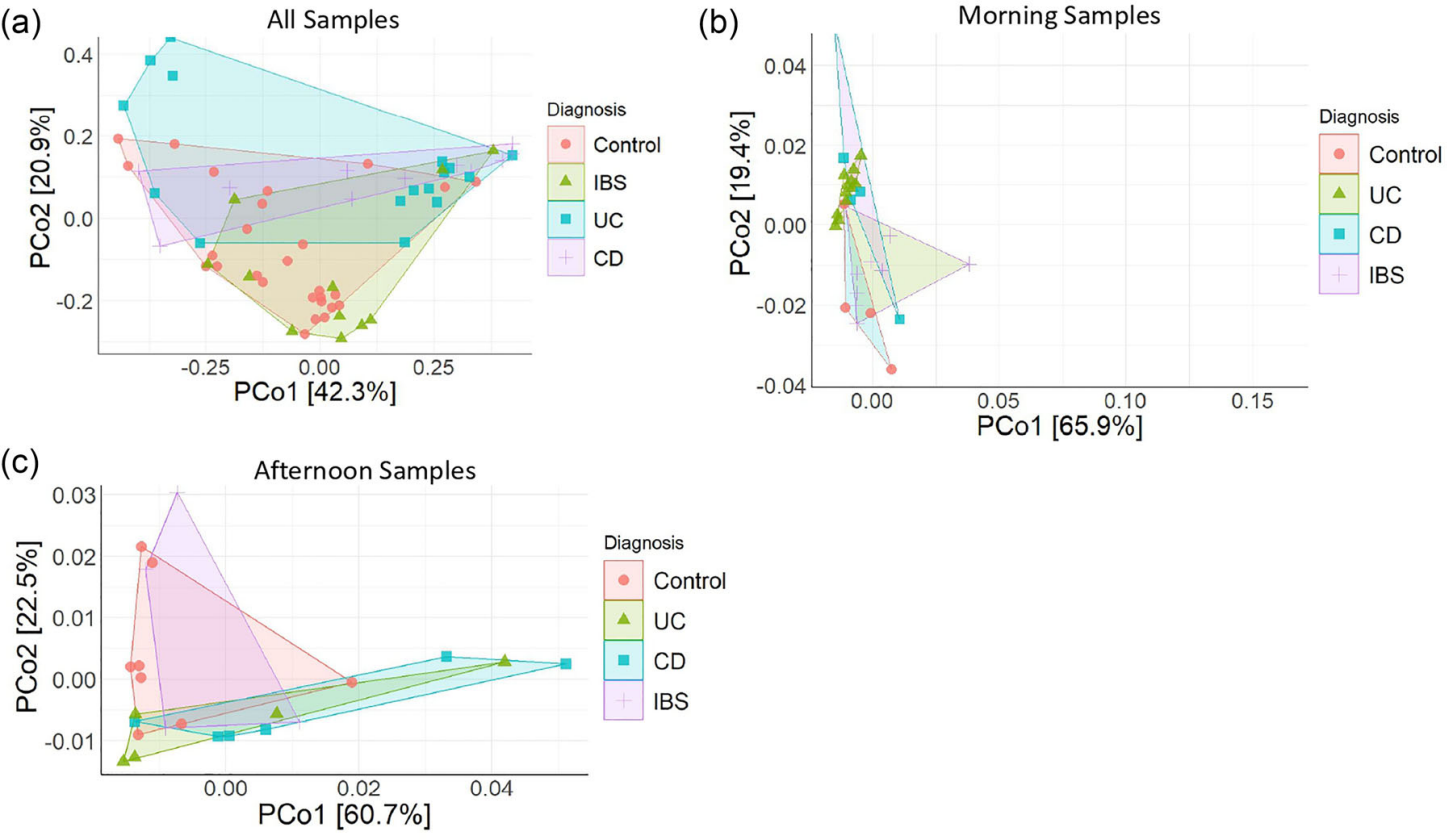
Analysis of circadian clock gene expression profiles stratified by morning and afternoon blood collection times. (a) Principal coordinate analysis plot showing the distribution of individual total circadian profiles across all study groups. *n* = 29 control subjects, *n* = 40 UC, *n* = 38 CD and *n* = 39 IBS. (b) PCoA plot showing the distribution of individual total circadian profiles from blood taken in the morning. *n* = 15 control subjects, *n* = 18 IBS, *n* = 16 UC, *n* = 18 CD. (c) PCoA plot showing the distribution of individual total circadian profiles from blood taken in the afternoon. *n* = 8 control subjects, *n* = 13 IBS, *n* = 11 UC and *n* = 12 CD. Abbreviations: CD, Crohn's disease; IBS, irritable bowel syndrome; PCo, principal coordinate; UC, ulcerative colitis.

**FIGURE 5:**
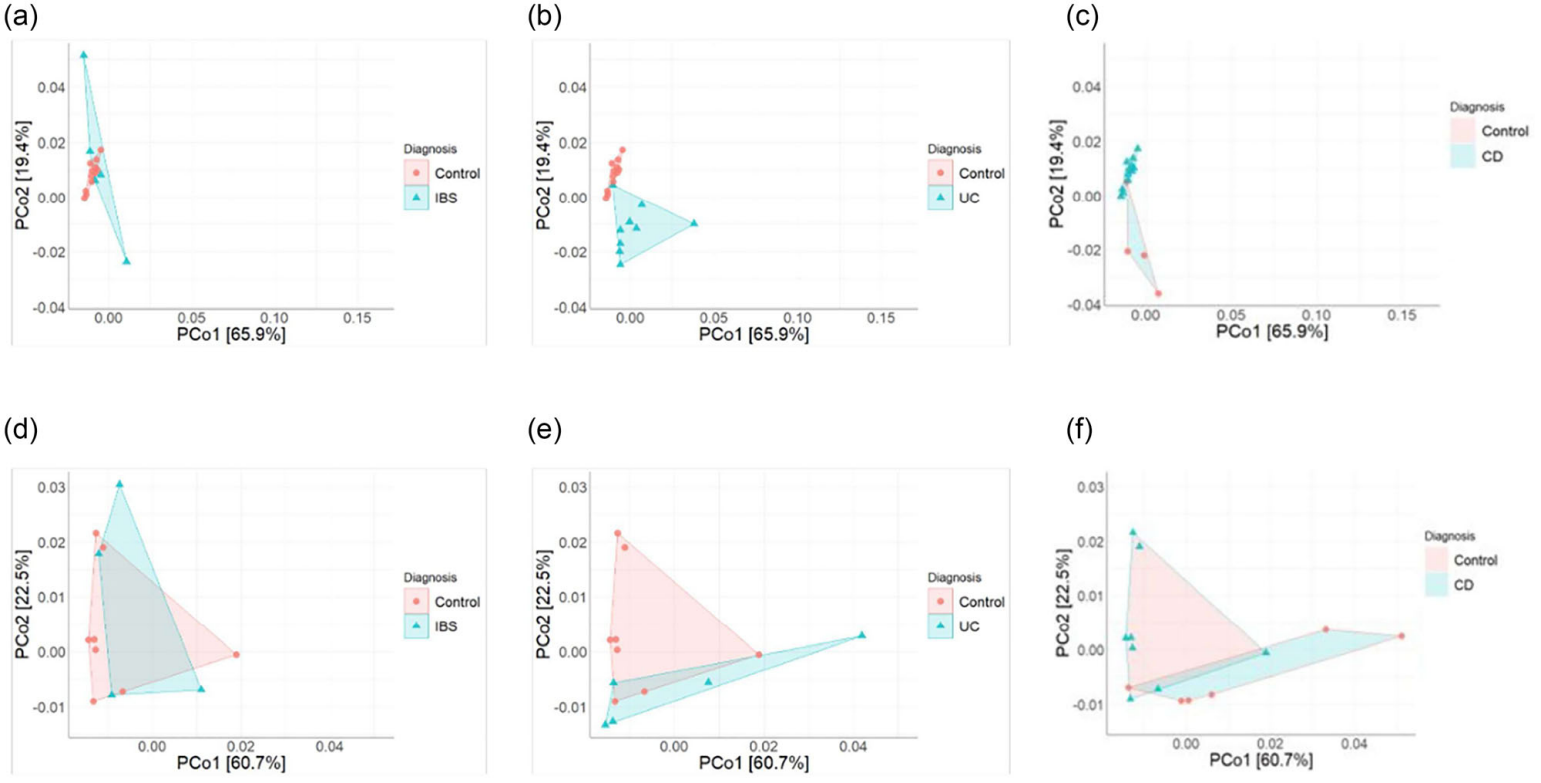
Representative breakdown of principal coordinate analysis data of clock gene expression. (a) Comparison of IBS and control subjects whose blood had been taken in the morning. (b) Comparison of UC and control subjects whose blood had been taken in the morning. (c) Comparison of CD and control subjects whose blood had been taken in the morning. *n* = 15 control subjects, *n* = 18 IBS, *n* = 16 UC and *n* = 18 CD. (d) Comparison of IBS and control subjects whose blood had been taken in the afternoon. (e) Comparison of UC and control subjects whose blood had been taken in the afternoon. (f) Comparison of CD and control subjects whose blood had been taken in the afternoon. Data are presented as the mean (SEM). *n* = 8 control subjects, *n* = 13 IBS, *n* = 11 UC and *n* = 12 CD. Abbreviations: CD, Crohn's disease; IBS, irritable bowel syndrome; PCo, principal coordinate; UC, ulcerative colitis.

To determine whether alterations in circadian gene expression were specific to individual GI conditions, quantitative PCR was used to determine the relative expression of *BMAL1*, *CLOCK*, *CRY1*, *CRY2*, *PER1*, *PER2*, *PER3*, *NR1D1* and *NR1D2*. To account for potential confounders within our cohort and variation in sample collection time, we performed a linear regression analysis to look at the effect of time of blood collection, age, sex and BMI on clock gene expression. There were no significant differences in *BMAL1*, *CLOCK*, *PER1*, *PER2*, *PER3*, *NR1D1* or *NR1D2* relative expression between control subjects and patients with GI disease (Figure 3; Table A2). However, *CRY1* relative expression was significantly higher in IBS compared with control subjects (*p *= 0.031) and in UC compared with control subjects (*p = *0.042) when controlling for the aforementioned factors (Figure 3c). Principal components analysis was then conducted to assess dissimilarities between groups based on all assayed clock genes, revealing no distinct separation in individual total circadian profiles between groups (Figure 4a). To help account for the effect of time of day on gene expression, PCoA was then performed on all the assayed clock genes split by sample collection time period. The samples taken in the morning (Figure 4b) and the afternoon (Figure 4c) showed no distinct separation in individual total circadian profile between groups (Figure 5). Collectively, these data indicate that *CRY1* gene expression is altered in IBD and IBS, irrespective of potential confounders, including time of sample collection.

### 3.5 Altered *NR1D1* and *NR1D2* gene expression between GI cohorts linked to fatigue

**FIGURE 6:**
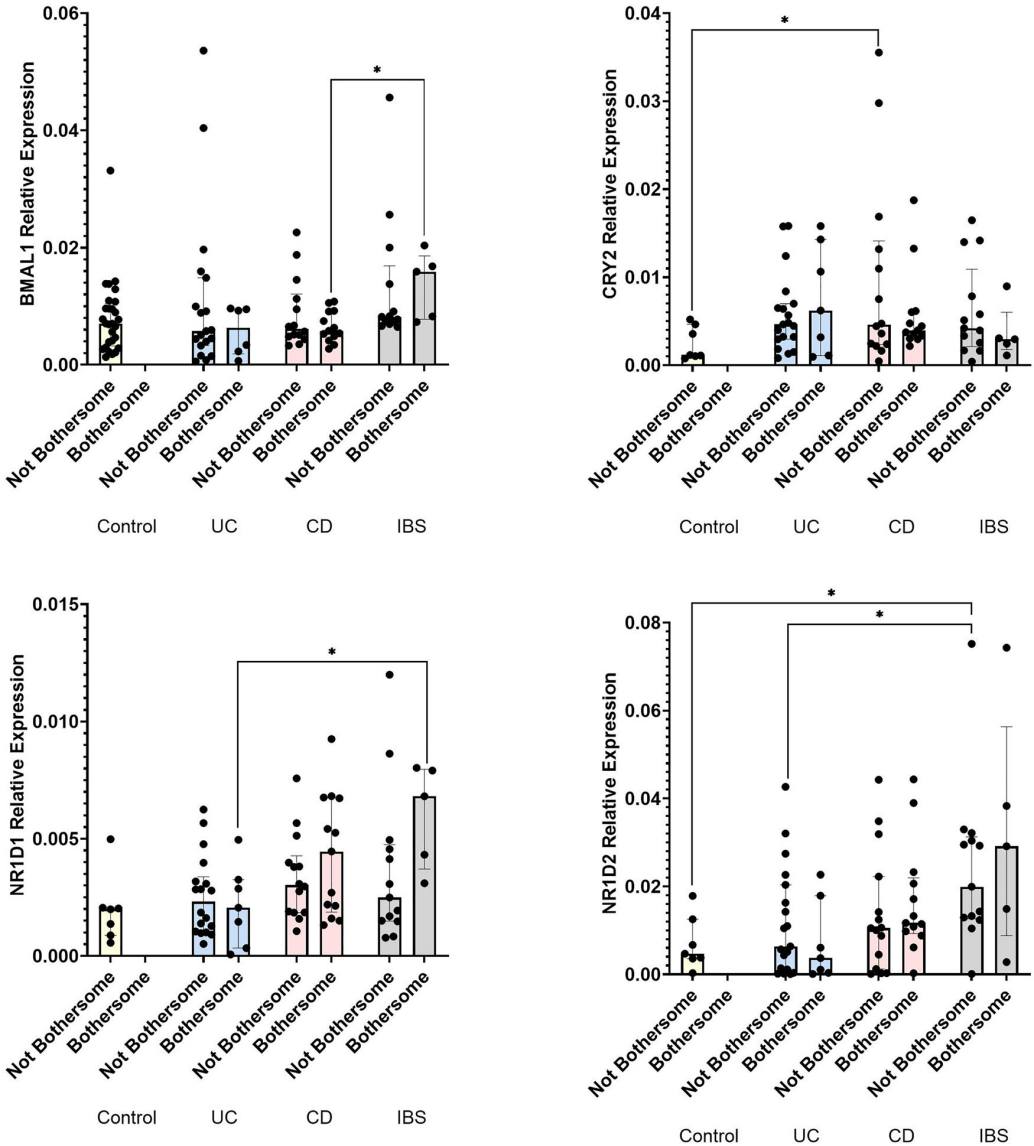
Circadian rhythmicity and fatigue. To analyse fatigue and circadian misalignment, clock gene expression was correlated with self‐reported fatigue in control subjects, UC, CD and IBS. Participants reported how bothersome the fatigue they experienced was, and quantitative PCR was used to analyse relative expression of *BMAL1* (a), *CRY2* (b), *Rev‐erbα* (c) and *Rev‐erbβ* (d) relative to the β‐actin housekeeping gene. *n* = 8 control subjects, *n* = 24 UC, *n* = 25 CD and *n* = 17 IBS. Data are presented as the median (interquartile range) and analysed by multiple Mann–Whitney *U*‐tests with multiple comparisons. *p < 0.05. Abbreviations: CD, Crohn's disease; IBS, irritable bowel syndrome; UC, ulcerative colitis.

To assess relationships between fatigue and the circadian system, clock gene expression was compared, separated by patient‐reported experience of fatigue. *BMAL1* relative expression was significantly higher in IBS participants reporting bothersome fatigue compared with CD (0.016 [0.019–0.008] IBS, 0.006 [0.009–0.005] CD, *p *= 0.019; Figure 6a; Table A3). There were no differences in *CLOCK*, *CRY1*, *PER1*, *PER2* or *PER3* expression in those who did not experience fatigue being bothersome between groups (Table A4). However, *CRY2* relative expression was significantly higher in UC participants who did not report fatigue being bothersome compared with control subjects (0.005 [0.007–0.003] UC, 0.001 [0.005–0.001] control, *p *= 0.041; Figure 6b). The relative expression of *NR1D1* was significantly higher in IBS participants who reported bothersome fatigue compared with UC patients with bothersome fatigue (0.007 [0.008–0.004] IBS, 0.002 [0.003–0.000] UC, *p *= 0.018; Figure 6c). Furthermore, *NR1D2* relative expression was significantly higher in IBS participants who did not experience bothersome fatigue compared with UC (0.020 [0.031–0.013] IBS, 0.006 [0.020–0.001] UC, *p *= 0.030) and control subjects without bothersome fatigue (*p *= 0.011; Figure 6d). These data suggest that the auxiliary feedback loop might be altered in GI disease and that this alteration might differentiate patients experiencing fatigue.

### 3.6 Increased *CRY1* relative expression is associated with sleep quality in CD

**FIGURE 7:**
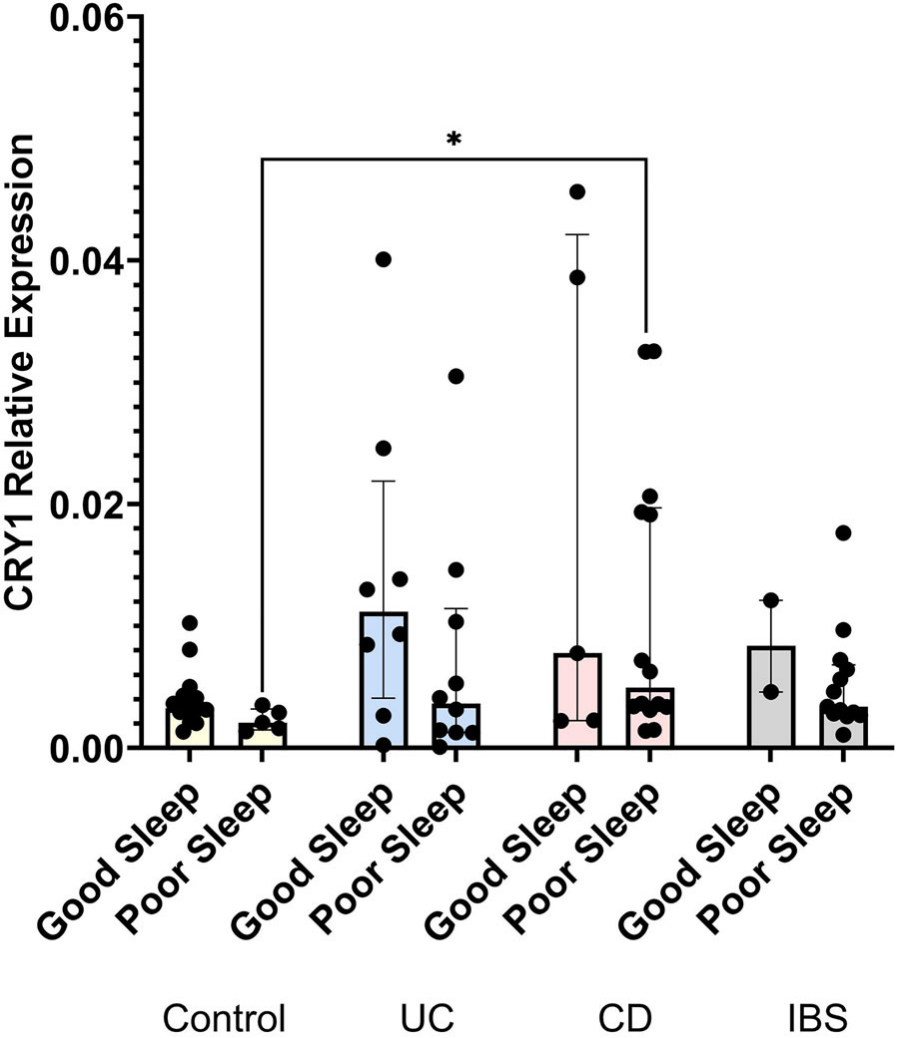
Circadian rhythmicity and sleep quality. To analyse sleep quality and circadian misalignment, clock gene expression was correlated with self‐reported sleep quality in control subjects, UC, CD and IBS. Participants reported how bothersome their sleep quality was, and quantitative PCR was used to analyse relative expression of *CRY1* as relative to the β‐actin housekeeping gene. *n* = 20 control subjects, *n* = 19 UC, *n* = 19 CD and *n* = 15 IBS. Data are presented as the median (interquartile range) and analysed by multiple Mann–Whitney *U*‐tests with multiple comparisons. *p < 0.05. Abbreviations: CD, Crohn's disease; IBS, irritable bowel syndrome; UC, ulcerative colitis.

To explore the relationship between sleep quality and circadian‐associated gene expression, gene expression was next compared separated by participants self‐reporting good and poor sleep quality. There were no significant differences in *BMAL1*, *CLOCK*, *CRY2*, *PER1*, *PER2*, *PER3*, *NR1D1* and *NR1D2* gene expression and sleep quality in IBD or IBS patients compared with control subjects (Tables A5 and A6). Interestingly, *CRY1* relative expression was significantly higher in CD participants reporting poor sleep quality compared with control subjects (0.005 [0.020–0.003] CD, 0.002 [0.003–0.002] control, *p* = 0.026; Figure 7). These data indicate that *CRY1* might be associated with or influence patients’ perception of sleep quality.

## 4 DISCUSSION

This was a retrospective study that aimed to determine whether there were alterations in peripheral clock gene expression in patients with GI conditions and whether these changes related to the sleep disturbance and fatigue that these patients commonly report. We observed increased rates of self‐reported fatigue and poorer sleep in IBS and IBD, consistent with previous reports (Chavarría et al., 2019; Jansen et al., 2021; Marinelli et al., 2020). Our main finding demonstrates that *CRY1* gene expression was significantly altered in GI disease, in comparison to community and outpatient control subjects, when time of collection and other confounders were accounted for. Specifically, alterations in the expression of *CRY1* might be associated with IBS and UC, which might explain why these patients experience more fatigue and poorer sleep than control subjects. However, there was high inter‐individual variability across *CRY1* expression, which could be considered reflective of the heterogeneous nature of these GI conditions. It is unclear what percentage of patients report their fatigue and sleep disruption preceding GI symptom onset or vice versa and whether this differentially affects circadian dysfunction, contributing further to the heterogeneity. Nevertheless, our findings support the hypothesis that a subset of patients’ symptoms might be influenced by the circadian system, with altered clock gene expression being associated with increased self‐reported sleep disturbance and fatigue in our GI disease cohorts.

Although *NR1D2* was increased in IBS compared with UC, this difference was non‐significant when accounting for sample collection time and other confounders. There is high variability between individuals, and rhythmicity of clock genes varies across sites; the PER genes are particularly rhythmic in peripheral blood mononuclear cells (Takata et al., 2002; Takimoto et al., 2005; Teboul et al., 2005), and *CRY1* exhibits varied rhythmicity in healthy individuals (Boivin et al., 2003).

Interestingly, *CRY1* was significantly increased in CD patients who slept poorly compared with control subjects in our cohort, and *CRY1* variants are associated with delayed sleep phase disorder (sleep onset is abnormally delayed in comparison to societal norms; Patke et al., 2017). Peripheral *CRY1* is also significantly reduced in UC patients with active disease, but it is comparable to control subjects in UC patients in remission, suggesting a potential relationship with inflammation in UC (Weintraub et al., 2023). As such, disease state is an important consideration in the interpretation of our findings. However, unfortunately, we were unable to determine the IBD disease state of participants at the time of sample collection owing to the retrospective nature of this study and the fact that samples from most participants were not collected at the time of endoscopy. Finally, *CRY1* is a negative regulator of *BMAL1*, with implications for regulation of circadian timing (Parico et al., 2020), suggesting that alteration of *CRY1* in GI disease might contribute to altered circadian timing and sleep patterns. Furthermore, when we separated gene expression based on morning and afternoon timing of blood collection, the GI disease groups show increased *NR1D2* compared with control subjects in both the morning and afternoon samples. This is interesting, because a previous study found that *NR1D2* gene expression was downregulated in patients with UC (Palmieri et al., 2015). Furthermore, one study reported that circadian patterns of heart rate variability are altered between states of active inflammation and remission in IBD patients (Hirten et al., 2025), indicating that the circadian system is altered depending on disease status. Given that we had no data on IBD location or disease status, this could account for the differences we saw in our data. Therefore, future research is required to understand fully the role of *NR1D2* expression in IBD pathophysiology.

Self‐reported data from sleep questionnaires demonstrated a higher rate of fatigue and worse sleep quality in IBS and IBD participants, supporting previous observations of increased incidence of fatigue and insomnia in both IBD and DGBI patients (Chavarría et al., 2019; Jansen et al., 2021; Marinelli et al., 2020). Sleep disturbances are also associated with an increased risk of DGBIs (Wang et al., 2018; Yamamoto et al., 2021), and interestingly, the chances of a DGBI patient reporting sleep trouble are increased in those who report more than one concomitant DGBI (Kim et al., 2018), suggesting that GI disease burden impacts circadian dysfunction. Unfortunately, only one IBS participant in this study reported concomitant DGBIs, meaning that we were unable to examine this further.

Overall, the literature suggests that sleep disturbances and fatigue are features of chronic GI disease, implicating the circadian system in the pathophysiology; however, the functional contribution to these abnormalities to symptom onset and severity or disease progression requires further investigation. Expression of *BMAL1*, *NR1D1* and *NR1D2* were significantly increased in the periphery of IBS patients compared with the IBD groups. This is interesting given that these genes are linked to the auxiliary feedback loop of the circadian system, which is involved in maintaining the daily rhythm (Yan et al., 2014). This finding might suggest that dysregulation of this loop is a feature of DGBIs, warranting further investigation. Furthermore, we found that those with IBS who reported experiencing bothersome fatigue had significantly higher *BMAL1* and *NR1D1* gene expression compared with the IBD cohort, whereas *NR1D2* expression was increased in IBS patients without bothersome fatigue, suggesting a relationship of *NR1D1* and *NR1D2* related to perception of fatigue. These findings support the notion that alterations to the auxiliary feedback loop might explain the increased fatigue and sleep disturbances experienced by patients with IBS. Given that the auxiliary feedback loop is involved in maintaining the robustness of the circadian system, one hypothesis might be that GI disease patients are more sensitive to circadian disruption, which contributes to symptom burden, given circadian regulation of immune activation and inflammation (Scheiermann et al., 2013). Therefore, studies investigating the correction of sleep quality as a therapeutic option are needed, because this might help to reduce visceral hypersensitivity and chronic symptoms experienced by these patients.

This study does have several limitations. Owing to the retrospective nature of the study, data for some participants were missing, including information on demographic characteristics, time of blood collections, and self‐reported data on sleep quality and fatigue. In addition, the questions capturing information on fatigue and sleep were brief, and shift work history was not collected. Future prospective cohort studies should consider more robust measures of these factors to assess associations between gene expression and sleep or fatigue better. Furthermore, blood samples were collected at varying times throughout the day, making it difficult to link specific gene expression profiles to time of day, and our results might be affected by the sampling time frame. Samples at different time points from the same individual were not taken, hence we were unable to assess the true impact of time of day on circadian gene expression to validate this finding. The implications suggest that future studies should consider the time of day of collection as a crucial factor in study design. Given that self‐reported data are not always reliable (Rosenman et al., 2011), this might further impact the interpretation of our findings. In addition, if participants have lived with fatigue, insomnia or other sleep disturbances for years, their perception of how this affects them might differ drastically from those for whom these issues are new or experienced infrequently, and this might contribute to the large variability between individuals. Owing to sample size limitation, we were unable to investigate clock gene expression between IBS subtypes; however, this is an important avenue of investigation in future, given that the underlying biology is likely to be different in patients with diarrhoeal and constipated phenotypes. Given that this was a retrospective analysis, only blood samples were available, but this condition is associated with food intake (a key regulator of the circadian system) for at least a subset of patients. GI tract‐specific clocks might exhibit unique patterns of gene expression owing to their specialized functions, direct exposure to nutrients and distinct cellular compositions, henceexamining gene expression in biopsies should be considered in future research. Thus, further well‐controlled studies are needed to demonstrate true differences in the circadian system in GI disease, particularly focusing on peripheral clocks located in the GI tract. Specific analysis of participants’ chronotypes and dim light melatonin onset will also provide further insight into participants’ endogenous circadian timing. However, we believe that the findings of this study are of importance because they demonstrate a clear difference in circadian gene expression between control subjects and IBS participants. Furthermore, to our knowledge, no other study has examined the expression of peripheral circadian genes in IBS in the context of self‐reported fatigue and sleep difficulties, hence the data are novel and suggest that this is an area worthy of further exploration in DGBIs.

## 5 CONCLUSION

In summary, our work reveals differences in clock gene expression profiles in whole blood, suggesting that *CRY1* gene expression is altered in IBS and IBD and might be associated with sleep quality. Given that this study was evaluating peripheral clock gene expression, the findings suggest that both organic and functional GI conditions have a distinct impact on systemic circadian rhythmicity, and future work might identify a predisposition to circadian abnormalities that contributes to susceptibility for GI disease. Importantly, the data suggest that *CRY1* might be related to the increased fatigue and poor sleep experienced by these patients.

## AUTHOR CONTRIBUTIONS

Sophie Fowler, Emily C. Hoedt, Grace L. Burns and Simon Keely participated in the design of the concept, hypothesis and aims of the study. Sophie Fowler and Grace L. Burns participated in the initial drafting of the manuscript. Sophie Fowler performed sample processing, gene expression experiments and analysis. Jennifer C. Pryor, Jessica K. Bruce, Cheenie Nieva and Prema M. Nair assisted with collection and processing of patient samples. Gemma M. Paech assisted with review of findings. Gemma M. Paech and Guy D. Eslick assisted with statistical analysis. Emily C. Hoedt and Simonne Sherwin provided technical support and assistance with protocols. Peter Pockney and Nicholas J. Talley assisted with participant recruitment and review of cohort. Nicholas J. Talley and Simon Keely provided resources. All authors read and approved the final version of the manuscript and agree to be accountable for all aspects of the work in ensuring that questions related to the accuracy or integrity of any part of the work are appropriately investigated and resolved. All persons designated as authors qualify for authorship, and all those who qualify for authorship are listed.

## CONFLICT OF INTEREST

S.F., J.C.P., J.K.B., C.N., P.M.N., E.C.H., S.S., G.L.B., G.M.P., G.D.E. and P.P.: None to disclose. N.J.T.: Non‐financial support from: Norgine (2021) (IBS interest group), personal fees from Allakos (gastroduodenal eosinophilic disease) (2021), Bayer (IBS) (2020), Planet Innovation (Gas capsule IBS) (2020), twoXAR Viscera Labs (USA 2021) (IBS‐diarrhoea), Dr Falk Pharma (2020) (EoE), Sanofi‐Aventis, Glutagen (2020) (coeliac disease), IsoThrive (2021) (oesophageal microbiome), BluMaiden (microbiome advisory board) (2021), Rose Pharma (IBS) (2021), Intrinsic Medicine (2022) (human milk oligosaccharide), Comvita Mānuka Honey (2021) (digestive health), Astra Zeneca (2022), outside the submitted work. In addition, Dr Talley has a patent Nepean Dyspepsia Index (NDI) 1998, Biomarkers of IBS licensed, a patent Licensing Questionnaires Talley Bowel Disease Questionnaire licensed to Mayo/Talley, a patent Nestec European Patent licensed, and a patent Singapore Provisional Patent ‘Microbiota Modulation of BDNF Tissue Repair Pathway’ issued, ‘Diagnostic marker for functional gastrointestinal disorders’ Australian Patent Application WO2022256861A1 via the University of Newcastle and UniQuest (University of Queensland). Dr Talley is supported by funding from the National Health and Medical Research Council (NHMRC) to the Centre for Research Excellence in Digestive Health and he holds an NHMRC Investigator grant. S.K.: Grants from National Health and Medical Research Council (Ideas Grant and Centre for Research Excellence), grants from Viscera Labs (Research contract), grants from Microba Life Science (Research contract), personal fees from Gossamer Bio, personal fees from Anatara Lifescience, personal fees from Immuron and personal fees from Microba Life Science.

## 1

**TABLE A1:** Experience of fatigue and bothersome fatigue (statistical data from Figure 2).

Parameter	Control	UC	CD	IBS	Control vs. UC (*p*‐value)	Control vs. CD (*p*‐value)	Control vs. IBS (*p*‐value)	UC vs. CD (*p*‐value)	UC vs. IBS (*p*‐value)	CD vs. IBS (*p*‐value)
Fatigue score, median [IQR]	1.00 [1.00–2.75]	3.00 [2.00–4.00]	3.00 [2.00–4.00]	2.00 [1.00–4.00]	0.187	0.080	>0.999	>0.999	>0.999	0.761
Bothersome fatigue score, median [IQR]	1.00 [0.25–1.00]	2.00 [1.00–2.00]	2.00 [1.00–4.00]	2.00 [1.00–3.00]	0.188	0.01	0.240	>0.999	>0.999	>0.999

Abbreviations: CD, Crohn's disease; IBS, irritable bowel syndrome; IQR, interquartile range; UC, ulcerative colitis.

**p *< 0.

**TABLE A2:** Circadian gene expression, controlling for age, sex, body mass index and time of blood collection (statistical data for Figure 3).

Gene	UC	CD	IBS
*BMAL1* (*R* ^2^, *p*‐value)	0.059, 0.919	0.165, 0.790	0.191, 0.118
*CLOCK* (*R* ^2^, *p*‐value)	0.102, 0.206	0.212, 0.790	0.169, 0.224
*CRY1* (*R* ^2^, *p*‐value)	0.165, 0.042*	0.239, 0.068	0.238, 0.031*
*CRY2* (*R* ^2^, *p*‐value)	0.079, 0.384	0.238, 0.068	0.136, 0.805
*PER1* (*R* ^2^, *p*‐value)	0.0594, 0.885	0.194, 0.249	0.171, 0.213
*PER2* (*R* ^2^, *p*‐value)	0.059, 0.945	0.199, 0.217	0.141, 0.614
*PER3* (*R* ^2^, *p*‐value)	0.059, 0.614	0.188, 0.217	0.135, 0.146
*Rev‐erbα* (*R* ^2^, *p*‐value)	0.066, 0.055	0.165, 0.497	0.183, 0.086
*Rev‐erbβ* (*R* ^2^, *p*‐value)	0.154, 0.924	0.174, 0.310	0.202, 0.887

Abbreviations: CD, Crohn's disease; IBS, irritable bowel syndrome; UC, ulcerative colitis.

* *p *< 0.05. Comparison is vs. control subjects.

**TABLE A3:** Clock gene expression and experience of bothersome fatigue (statistical data from Figure 5).

Gene	Control	UC	CD	IBS	UC vs. CD (*p*‐value)	UC vs. IBS (*p*‐value)	CD vs. IBS (*p*‐value)
*BMAL1*, median [IQR]	–	0.006 [0.010–0.002]	0.006 [0.009–0.005]	0.016 [0.019–0.008]	0.701	0.126	0.019*
*CLOCK*, median [IQR]	–	0.004 [0.017–0.001]	0.006 [0.011–0.003]	0.008 [0.017–0.005]	0.711	0.268	0.279
*CRY1*, median [IQR]	–	0.003 [0.015–0.001]	0.003 [0.014–0.001]	0.005 [0.008–0.003]	0.939	0.876	0.702
*CRY2*, median [IQR]	–	0.006 [0.014–0.001]	0.004 [0.006–0.003]	0.003 [0.006–0.002]	0.817	0.432	0.117
*PER1*, median [IQR]	–	0.003 [0.005–0.002]	0.003 [0.005–0.002]	0.004 [0.007–0.002]	0.877	0.530	0.633
*PER2*, median [IQR]	–	0.004 [0.008–0.000]	0.004 [0.005–0.002]	0.002 [0.009–0.001]	0.877	0.876	0.289
*PER3*, median [IQR]	–	0.001 [0.001–0.000]	0.001 [0.001–0.001]	0.001 [0.002–0.001]	0.183	0.202	0.775
*Rev‐erbα*, median [IQR]	–	0.002 [0.003–0.000]	0.004 [0.007–0.002]	0.007 [0.008–0.004]	0.081	0.018*	0.173
*Rev‐erbβ*, median [IQR]	–	0.004 [0.018–0.000]	0.012 [0.022–0.009]	0.029 [0.056–0.009]	0.081	0.073	0.289

*Note*: Control subjects did not experience bothersome fatigue, hence correlations between disease groups and control subjects were not performed.

Abbreviations: CD, Crohn's disease; IBS, irritable bowel syndrome; IQR, interquartile range; UC, ulcerative colitis.

* *p *< 0.05.

**TABLE A4:** Clock gene expression and no experience of bothersome fatigue (statistical data from Figure 5).

Gene	Control	UC	CD	IBS	Control vs. UC (*p*‐value)	Control vs. CD (*p*‐value)	Control vs. IBS (*p*‐value)	UC vs. CD (*p*‐value)	UC vs. IBS (*p*‐value)	CD vs. IBS (*p*‐value)
*BMAL1*, median [IQR]	0.007 [0.010–0.003]	0.006 [0.015–0.003]	0.006 [0.012–0.005]	0.008 [0.017–0.007]	0.906	0.742	0.108	0.634	0.108	0.061
*CLOCK*, median [IQR]	0.003 [0.006–0.002]	0.007 [0.015–0.003]	0.007 [0.036–0.003]	0.006 [0.008–0.003]	0.090	0.055	0.151	0.343	0.545	0.347
*CRY1*, median [IQR]	0.003 [0.008–0.002]	0.004 [0.010–0.001]	0.005 [0.033–0.002]	0.006 [0.010–0.003]	0.836	0.267	0.241	0.244	0.275	>0.999
*CRY2*, median [IQR]	0.001 [0.005–0.001]	0.005 [0.007–0.003]	0.005 [0.014–0.002]	0.004 [0.011–0.002]	0.041*	0.067	0.097	0.679	>0.999	0.756
*PER1*, median [IQR]	0.002 [0.003–0.001]	0.002 [0.003–0.001]	0.002 [0.008–0.001]	0.002 [0.003–0.001]	0.836	0.447	0.726	0.630	0.471	0.300
*PER2*, median [IQR]	0.003 [0.004–0.001]	0.002 [0.006–0.001]	0.003 [0.010–0.002]	0.003 [0.007–0.001]	0.790	0.322	0.938	0.193	0.708	0.302
*PER3*, median [IQR]	0.002 [0.003–0.001]	0.001 [0.002–0.001]	0.001 [0.002–0.001]	0.001 [0.003–0.001]	0.657	0.891	0.938	0.464	0.567	0.928
*Rev‐erbα*, median [IQR]	0.002 [0.002–0.001]	0.002 [0.003–0.001]	0.003 [0.004–0.002]	0.002 [0.005–0.001]	0.495	0.110	0.351	0.156	0.465	0.685
*Rev‐erbβ*, median [IQR]	0.005 [0.013–0.004]	0.006 [0.020–0.001]	0.011 [0.022–0.001]	0.020 [0.031–0.013]	0.651	0.490	0.011*	0.707	0.030*	0.080

Abbreviations: CD, Crohn's disease; IBS, irritable bowel syndrome; IQR, interquartile range; UC, ulcerative colitis.

* *p *< 0.05.

**TABLE A5:** Clock gene expression and good sleep quality (statistical data from Figure 6).

Gene	Control	UC	CD	IBS	Control vs. UC (*p*‐value)	Control vs. CD (*p*‐value)	Control vs. IBS (*p*‐value)	UC vs. CD (*p*‐value)	UC vs. IBS (*p*‐value)	CD vs. IBS (*p*‐value)
*BMAL1*, median [IQR]	0.010 [0.014–0.007]	0.010 [0.015–0.006]	0.011 [0.021–0.006]	0.015 [0.016–0.014]	0.971	0.823	0.200	0.876	0.333	0.857
*CLOCK*, median [IQR]	0.007 [0.008–0.004]	0.010 [0.017–0.004]	0.012 [0.039–0.003]	0.005 [0.008–0.003]	0.188	0.391	0.817	0.622	0.400	0.381
*CRY1*, median [IQR]	0.003 [0.004–0.003]	0.011 [0.022–0.004]	0.008 [0.042–0.002]	0.008 [0.012–0.005]	0.050	0.343	0.100	0.943	0.711	>0.999
*CRY2*, median [IQR]	0.005 [0.007–0.004]	0.007 [0.012–0.005]	0.013 [0.024–0.003]	0.008 [0.009–0.008]	0.131	0.266	0.059	0.622	0.711	0.857
*PER1*, median [IQR]	0.003 [0.004–0.002]	0.003 [0.004–0.001]	0.006 [0.008–0.001]	0.004 [0.007–0.001]	0.616	0.444	>0.999	0.354	>0.999	0.857
*PER2*, median [IQR]	0.005 [0.006–0.003]	0.005 [0.007–0.002]	0.010 [0.016–0.002]	0.008 [0.014–0.002]	0.925	0.266	0.941	0.435	0.711	>0.999
*PER3*, median [IQR]	0.002 [0.002–0.001]	0.002 [0.003–0.001]	0.002 [0.003–0.001]	0.002 [0.003–0.001]	0.591	0.866	0.824	>0.999	0.889	>0.999
*Rev‐erbα*, median [IQR]	0.005 [0.006–0.002]	0.003 [0.005–0.003]	0.005 [0.007–0.002]	0.005 [0.007–0.003]	0.265	>0.999	0.824	0.622	0.533	0.857
*Rev‐erbβ*, median [IQR]	0.018 [0.026–0.004]	0.006 [0.025–0.000]	0.009 [0.027–0.000]	0.025 [0.029–0.020]	0.325	0.395	0.294	0.833	0.267	0.381

Abbreviations: CD, Crohn's disease; IBS, irritable bowel syndrome; IQR, interquartile range; UC, ulcerative colitis.

**TABLE A6:** Clock gene expression and poor sleep quality (statistical data from Figure 6).

Gene	Control	UC	CD	IBS	Control vs. UC (*p*‐value)	Control vs. CD (*p*‐value)	Control vs. IBS (*p*‐value)	UC vs. CD (*p*‐value)	UC vs. IBS (*p*‐value)	CD vs. IBS (*p*‐value)
*BMAL1*, median [IQR]	0.007 [0.010–0.002]	0.009 [0.016–0.004]	0.008 [0.010–0.005]	0.009 [0.019–0.007]	0.441	0.566	0.095	0.691	0.494	0.058
*CLOCK*, median [IQR]	0.005 [0.007–0.002]	0.006 [0.019–0.002]	0.007 [0.028–0.006]	0.008 [0.010–0.004]	0.267	0.095	0.160	0.494	0.976	0.574
*CRY1*, median [IQR]	0.002 [0.003–0.002]	0.004 [0.011–0.001]	0.005 [0.020–0.003]	0.003 [0.007–0.003]	0.594	0.026*	0.075	0.212	0.830	0.202
*CRY2*, median [IQR]	0.003 [0.005–0.001]	0.005 [0.006–0.001]	0.004 [0.013–0.003]	0.003 [0.006–0.002]	0.441	0.095	0.633	0.494	0.955	0.169
*PER1*, median [IQR]	0.003 [0.005–0.001]	0.002 [0.003–0.001]	0.003 [0.009–0.002]	0.002 [0.004–0.001]	0.679	0.559	0.646	0.235	0.628	0.176
*PER2*, median [IQR]	0.004 [0.006–0.002]	0.002 [0.007–0.001]	0.005 [0.007–0.003]	0.002 [0.006–0.001]	0.594	0.503	0.566	0.148	0.879	0.153
*PER3*, median [IQR]	0.001 [0.002–0.001]	0.001 [0.002–0.001]	0.001 [0.002–0.001]	0.001 [0.003–0.000]	0.767	0.823	0.849	0.585	0.750	0.793
*Rev‐erbα*, median [IQR]	0.002 [0.006–0.001]	0.003 [0.005–0.001]	0.004 [0.007–0.002]	0.004 [0.006–0.002]	0.953	0.289	0.503	0.257	0.376	0.920
*Rev‐erbβ*, median [IQR]	0.013 [0.019–0.006]	0.006 [0.018–0.000]	0.012 [0.023–0.009]	0.019 [0.030–0.011]	0.583	0.964	0.289	0.317	0.082	0.155

Abbreviations: CD, Crohn's disease; IBS, irritable bowel syndrome; IQR, interquartile range; UC, ulcerative colitis.

* *p *< 0.05.
